# NEMESISdb: A full length 16S rRNA gene dataset for the detection of human, fish, and crustacean potentially pathogenic bacteria

**DOI:** 10.1016/j.dib.2025.112135

**Published:** 2025-10-06

**Authors:** Son-Hoang Tran, Claudia Ximena Restrepo-Ortiz, Dinh Quang Vu, Marc Troussellier, Yvan Bettarel, Thierry Bouvier, Van Ngoc Bui, Nguyen Hieu Minh, Trung Du Hoang, Quang Huy Nguyen, Jean-Christophe Auguet

**Affiliations:** a UMR MARBEC, Univ Montpellier, CNRS, Ifremer, IRD, Montpellier, France; b University of Science and Technology of Hanoi, Vietnam Academy of Science and Technology (VAST), 18 Hoang Quoc Viet, Hanoi, Vietnam; c UMR MARBEC, Univ Montpellier, CNRS, Ifremer, IRD, Sète, France; d Institute of Biology (IB), Vietnam Academy of Science and Technology (VAST), 18 Hoang Quoc Viet, Hanoi, Vietnam; e Institute of Oceanography, Vietnam Academy of Science and Technology (VAST), 01, Cau Da, Nha Trang, Khanh Hoa, Vietnam; f Graduate University of Science and Technology (GUST), Vietnam Academy of Science and Technology (VAST), 18 Hoang Quoc Viet, Hanoï, Vietnam

**Keywords:** One Health, Dataset, Pathogenic bacteria, Marine ecosystems, Human, Animal

## Abstract

NEMESISdb is a 16S rRNA full length sequence curated dataset designed to enable the identification and tracking of potentially pathogenic bacteria (PPB) for human, fish, and crustacean hosts. It addresses the limited focus on marine and coastal environments as key reservoirs for PPB, where bacteria from diverse sources—terrestrial, marine, and animal—can coexist. Leveraging recent advances in high-throughput sequencing, NEMESISdb provides a robust resource for the detection of PPB in 16S rRNA gene metabarcoding or metagenomic data. The database comprises three datasets corresponding to human, fish, and crustacean hosts, containing 1703, 222, and 64 PPB species, respectively, with a total of over 150,000 16S rRNA full length sequences curated for accuracy. This resource was constructed by extracting sequences from the SILVA 138.2 SSU Ref NR99 database, refining them through a rigorous curation pipeline to ensure taxonomic consistency and eliminate misclassifications. The resulting datasets are optimized for use with popular tools such as BLAST and classifier software, enabling rapid and accurate detection of PPB in metabarcoding and metagenomic data. NEMESISdb supports diverse applications, including pathogen surveillance in aquatic ecosystems, studies on environmental factors influencing PPB dynamics, and the development of targeted strategies for mitigating pathogen impacts in aquaculture. Additionally, it facilitates research within the One Health framework by linking the circulation of PPB across environmental, animal, and human compartments.

Specifications Table

**Table:** 

Subject	Microbiology
Specific subject area	Full length 16S rRNA gene sequences pathogenic bacteria dataset
Type of data	Information table, Pathogens lists, Filtered fasta files, Python scripts
Data collection	We constructed a list of pathogenic bacteria for humans, fishes, and crustaceans from various studies and pathogen detection pipeline such as 16SPIP, FAPROTAX, MPD and MBPD. Afterward, full length 16S rRNA gene sequences of each of the pathogenic bacteria of the list was downloaded from the SILVA 138.2 SSU Ref NR99 bacterial database in order to obtain three pathogenic reference datasets for humans, fishes, and crustaceans, respectively. Lastly, each dataset was curated with homemade scripts to remove all sequences wrongly assigned at the species taxonomic level in SILVA 138.2 SSU Ref NR99.
Data source location	Raw data for the construction of the pathogen list came from Zhang et al. [1], Zhang et al. [2] Wardeh et al. [3], Blauwkamp et al. [4], Urban et al. [5], Louca et al. [6], Miao et al. [7] and Xinrun et al. [8]. Full length 16S rRNA gene sequences of each of the pathogenic bacteria of the list were downloaded from the SILVA 138.2 SSU Ref NR99 bacterial database [9].
Data accessibility	Repository name: Zenodo Data identification number: 10.5281/zenodo.16992968 Direct URL to data: https://doi.org/10.5281/zenodo.16992968
Related research article	None

## 1 Value of the Data

• NEMESISdb is a set of three curated 16S rRNA full length sequence datasets enabling the identification and tracking of potentially pathogenic bacteria (PPB) across human, fish and crustacean hosts and helping reveal factors that influence their dynamics.
• NEMESISdb can be directly and easily used in blast or in classifier softwares for fast detection of PPB in 16S rRNA gene metabarcoding or metagenomic data.
• NEMESISdb could benefit a wide range of stakeholders involved in diseases outbreak prevention and food security (e.g. health agencies, aquaculture and fisheries industries), biodiversity conservation and pathoecology (e.g. researchers and environmental monitoring organizations) and coastal management (e.g. policy makers).
• These datasets can be utilized and reused in several ways to provide further insights in pathogen surveillance by monitoring the dynamics and hotspot of PPB in aquatic environments, in comparative studies aiming to investigate how environmental factors influence pathogen diversity and abundance, in targeted interventions and mitigation strategies by guiding aquaculture management practices, to reduce pathogen impact and in the framework of One Health studies by facilitating the identification of PPB circulating within the environmental, animal and human compartments.

## 2 Background

Most research on infection diseases has focused on inland systems with comparatively little efforts directed towards marine habitats. However, marine and particularly coastal environments can function as transmission foci for potentially pathogenic bacteria (PPB) because of the concentrated aggregations of bacteria from different sources, both marine and terrestrial, where environmental, human, and/or animal related bacteria can coexist [10,11]. Comprehensive pathogen monitoring in water is difficult to achieve using commonly applied approaches, such as culture-based techniques or quantitative polymerase chain reaction (qPCR), due to their limited throughput [12]. Recent breakthroughs in high-throughput sequencing technologies now allow for the detection of PPB on an unprecedented scale using 16S rRNA gene sequencing [[13], [14], [15], [16], [17], [18]]. The accuracy and breadth of pathogen detection through 16S sequencing largely depend on the reference pathogen database used [8]. However, the datasets needed to precisely identify PPB circulating among the human, marine environment and marine animal compartments accordingly to a One Health framework remain largely underdeveloped. Here, we constructed NEMESISdb, a set of three curated 16S rRNA full-length sequence datasets, allowing the use of both long-read and short-read sequencing across different 16S rRNA gene variable regions to accurately detect PPB. NEMESISdb is a convenient tool for the rapid identification of human, fish, and crustacean PPB in next generation sequencing (NGS) data, supporting key areas such as food safety, epidemic prevention in both livestock and humans, disease detection, and environmental surveillance*.*

## 3 Data Description

NEMESISdb [9], available with the following DOI 10.5281/zenodo.16992968, is composed of 14 files and one folder. These include three fasta files containing the full-length 16S rRNA gene sequences of human, fish, and crustacean datasets; three tab-separated text files listing the genus–species pairs of PPB used to construct each dataset; one Excel file providing information on the sources used; and, for each group, an Excel file giving the taxonomic synonyms identified as well as another Excel file listing the species that compose the curated datasets together with their corresponding synonyms. In addition, a GitHub repository is provided containing the PathoLens Python package used to create and curate the datasets. Finally, we provide also in the zip file named “PPB_not_dereplicated.zip” three additional fasta files containing the full-length 16S rRNA gene sequences of human, fish, and crustacean datasets resulting from the application of the PathoLens Python package on the SILVA 138.2 SSU Ref database.

The three files, Human_Pathogen_DB.fasta, Fish_Pathogen_DB.fasta, Crustacean_Pathogen_DB.fasta contain the full-length 16S rRNA gene sequence of PPB for humans, fishes and crustacean respectively. Headers of each sequence within the fasta files correspond to the ACC number followed by the SILVA 138.2 SSU Ref NR99 taxonomy of the sequence from the kingdom to the species level. The datasets contain 8 795, 20 849 and 50 973 16S rRNA gene sequences with an average length of 1479.1 bp, 1491.3 bp, and 1499.4 bp, respectively for crustaceans, fishes and human (Table 1). This number of sequences encompasses 64, 222 and 1703 species of PPB for crustaceans, fishes and human, respectively.

**Table 1:** Summary of dataset’s properties for PPB retrieved from the SILVA 138.2 SSU Ref NR99 database.

	Crustacean	Fish	Human
Species	64	222	1703
Sequences	8795	20,849	50,973
Length's mean (bp)	1479.1	1491.3	1499.4
Length's sd (bp)	89.5	84.1	79.4

**Fig. 1:**
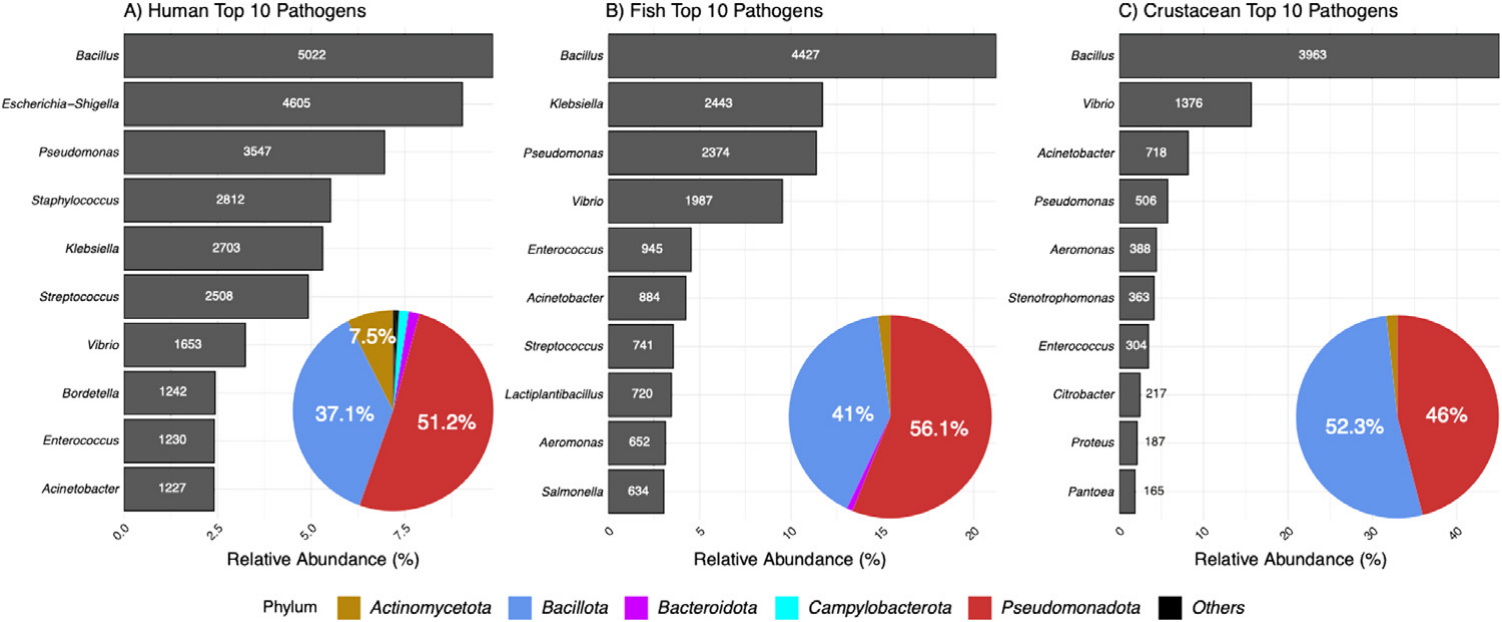
Taxonomic composition of the three PPB datasets. Barplot represents the contribution of the top ten genera in each dataset. The number of full length 16S rRNA gene sequences in each genus is indicated. Pie chart represents the taxonomic composition of each dataset at the phylum level (The percentage of Phyla higher than 5 % is indicated).

Overall, PPB sequences from the three datasets mainly belonged to the same two phyla namely *Bacillota* and *Pseudomonadota*, which represented on average 50.83 % and 42.66 % of the PPB dataset (Fig. 1). The diversity of PPB sequences was greater in humans, with twelve phyla represented, compared to four and three phyla observed in fishes and crustaceans, respectively. *Bacillus* was the most represented genus in the three datasets and represented up to 45 % of all the PPB sequences in the crustacean dataset. Similarly to *Bacillus*, other genera such as *Pseudomonas, Vibrio, Enterococcus* and *Acinetobacter* were common to the three datasets. As expected, we observed also some differences of composition among the 10 most represented genera of each dataset with notably the presence of *Aeromonas* only in fishes and crustacean datasets while the genera *Escherichia-Shigella, Staphylococcus* and *Bordella* were only present in the human dataset.

The “Pathogen_dataset_sources.xls” file contains 2 sheets indicating the different sources where the PPB derived from (sheet 1) and the list of PPB species extract from each source (sheet 2).

The initial list of PPB species used to generate extended list and extract the full length 16S rRNA gene sequences from the (non redundant) SILVA 138.2 SSU Ref NR99 database is contained in three tab-separated text files containing the genus-species pairs of PPB for each host group: Crustacean_sp_pathogens_list.txt (70 species), Fish_sp_pathogens_list.txt (240 species), and Human_sp_pathogens_list.txt (1942 species)

The Zenodo repository contains a GitHub repository of the PathoLens package, a Python tool designed to filter and curate taxonomic databases. It includes various modules and functions for validating records, which were used in the creation of the three PPB datasets.

Since overrepresentation of sequences in reference databases can impact the accuracy and precision of taxonomy assignment in rRNA studies [19], the three files, Human_Pathogen_DB.fasta, Fish_Pathogen_DB.fasta, Crustacean_Pathogen_DB.fasta have been obtained by applying our PathoLens Python package on the non redundant SILVA 138.2 SSU Ref NR99. However, we also provide an additional zip file in the Zenodo repository, containing the datasets obtained on the complete SILVA 138.2 SSU Ref database. This is important because certain PPB species or strains share >99 % identity across the full length of their 16S rRNA gene. Such strains are eliminated during dereplication in the SILVA 138.2 SSU Ref NR99 database, which can artificially reduce the apparent richness of the PPB community (Table 2). Moreover, when using classifiers on the non-redundant dataset, these species or strains may be subject to over-classification.

**Table 2:** Summary of dataset’s properties for PPB retrieved from the complete SILVA 138.2 SSU Ref database.

	Crustacean	Fish	Human
Species	65	223	1757
Sequences	34,481	80,761	196,770
Unique sequences	26,670	57,663	115,991
Length's mean (bp)	1150.7	1151.8	1158.8
Length's sd ((bp)	63.6	66.3	65.7

## 4 Experimental Design, Materials and Methods

### 4.1 Data acquisition and cleaning

To support the tracking and identification of potentially pathogenic bacteria (PPB) across different hosts, we developed PathoLens v0.1 [20], a custom Python 3.10.9 package tailored for this study. PathoLens integrates modular scripts and functions for automated data retrieval, processing, and curation of reference sequences. The package includes configuration files that define all required dependencies, ensuring reproducibility and ease of use.

The primary focus of this work was to build a curated set of 16S rRNA datasets enabling the tracking of potentially pathogenic bacteria (PPB) across hosts and their rapid detection using BLAST [21] or classifier software. The human PPB list was constructed using a list of pathogenic bacteria for humans from various studies [[2], [3], [4], [5]] and pathogen detection pipeline such as 16SPIP [7], FAPROTAX [6], MPD [1] and MBPD [8] (See “Pathogen_dataset_sources.xls” file for details). The fish and crustacean PPB lists were derived from the study of Wardeh et al. [3]. Crustacean PPB were not explicitly listed in the Wardeh dataset but were grouped under arthropods. To isolate crustacean pathogens, we used the script “ensembl_crustacea.py”, included in the PathoLens package. This script queries the Ensembl REST API [22], a comprehensive genome browser that provides various tools such as BLAST, BLAT [23], BioMart [24], and the Variant Effect Predictor (VEP) for all supported species. The script was designed to check if a given species belongs to the Crustacea class, by querying the Ensembl database for taxonomic information and determines whether the species falls under the "Crustacea" class. If it does, the species is labeled as a crustacean in the output. The script reads the input CSV file [Dataset] “SpeciesInteractions_EID.csv”, which contains information on host-pathogen interactions [3,25]. Once the list of PPB for humans, fish, and crustaceans was obtained, three tab-separated text files containing the genus-species pairs of PPB for each host group: “Crustacean_sp_pathogens_list.txt”, “Fish_sp_pathogens_list.txt” and “Human_sp_pathogens_list.txt” were prepared for further analysis.

Given the dynamic nature of bacterial taxonomy and the fact that databases such as SILVA are not updated synchronously with taxonomic databases like NCBI Taxonomy [26], we performed a thorough synonym search for each genus-species pair in these intermediate lists to maximize sequence recovery. This was done using the script get_sp_synonyms.py, which queries the NCBI Taxonomy database via Bio.Entrez package from Biopython [27]. For each species name, the script retrieves its currently accepted scientific name along with all known synonyms. In cases where no taxonomic record was found, the script performs a secondary search in the general NCBI database to obtain an accession number—provided the entry is valid and not associated with uncultured or unknown organisms—and uses it to retrieve the correct taxonomic ID and associated name. This process yields an expanded taxonomy that includes all known naming variants for each species. The script generates an Excel file per host group (CRUSTACEAN_Pathogen_TaxSyn_List.xlsx, FISH_Pathogen_TaxSyn_List.xlsx, HUMAN_Pathogen_TaxSyn_List.xlsx) that lists all taxonomic variants (synonyms, basionyms and ‘included’ names) identified for each pathogenic species. From this, an intermediate file is created with the extended species list including all nomenclatural variants for further query of the SILVA 138.2 SSU Ref NR99 database (CRUSTACEAN_sp_pathogens_list-EXT.txt, FISH_sp_pathogens_list-EXT.txt, HUMAN_sp_pathogens_list-EXT.txt), and a curated list of pathogenic species containing only the currently accepted scientific names, which serves as the final reference for each host group.

### 4.2 Generate SILVA reference pathogens dataset

**Fig. 2:**
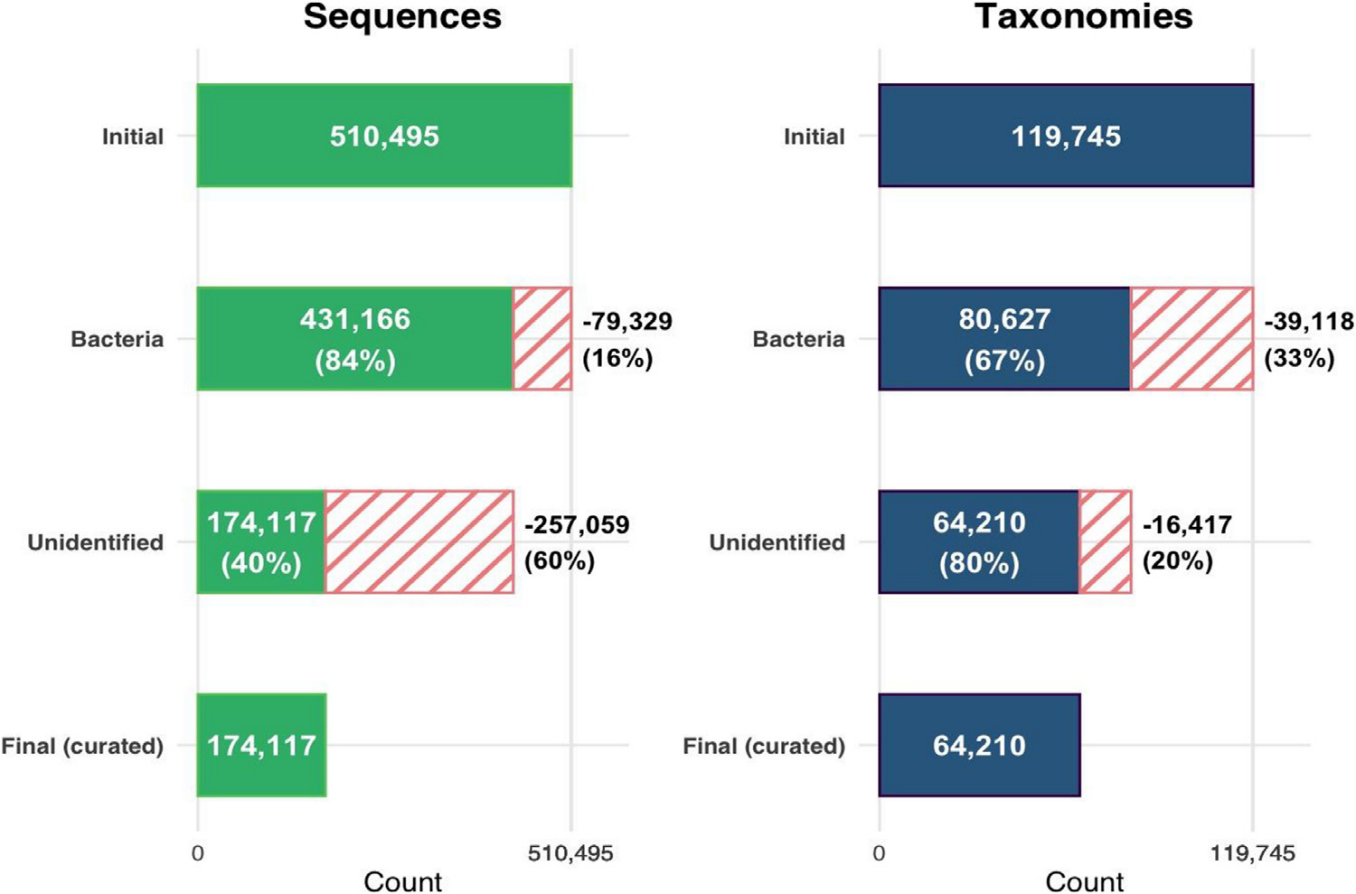
Overview of sequence and taxonomy retention across data cleaning steps in the construction of the SILVA reference pathogen dataset. Bars represent the total number of entries retained (solid) and removed (striped) at each stage of the pipeline: Initial, Bacteria filtering, Unidentified/uncultured removal, and Final (curated). For each step, the number and percentage of retained and removed entries are indicated. The left panel shows the evolution of sequence entries, and the right panel displays unique taxonomies.

To generate the SILVA reference pathogen dataset, the *database_builder* module (“1_run_database_builder.py”) from the PathoLens package was implemented. The process began by filtering the SILVA 138.2 SSU Ref NR99 database to retain only entries corresponding to the taxon Bacteria. At this step, 15.53 % (79 329 sequences) of the initial sequences and 32.67 % (39 118 taxonomies) of the unique taxonomies (i.e.; identical taxonomy from the kingdom to the species level) were excluded. Next, all sequences labeled as “uncultured,” “unidentified,” “unclassified,” “uncultivated,” “unculturable,” or “unicellular” were systematically removed to ensure the quality and relevance of the data. At this step, 59.61 % (257 059 sequences) of the Bacteria sequences and 20.36 % (16 417 taxonomies) of the unique taxonomies were excluded. After cleaning, the “Bacteria_filtered.fasta” dataset was created and used to extract species matches from the extended PPB species list generated in prior steps. These matches were cross-referenced with the Bacteria dataset for each host group, ensuring that only relevant pathogens were included. Finally, a custom pathogen dataset was generated for each host group (CRUSTACEAN_Pathogen_DB_Unfiltered.fasta, FISH_Pathogen_DB_Unfiltered.fasta and HUMAN_Pathogen_DB_Unfiltered.fasta), which will serve as the basis for the subsequent steps in the analysis pipeline. Most filtering occurred during the removal of unidentified or uncultured entries, resulting in the exclusion of over 257,000 sequences and 16,000 taxonomies, (Fig. 2).

### 4.3 Data curation

After extracting the sequences from the SILVA 138.2 SSU Ref NR99 database, a comprehensive curation process was applied to each FASTA dataset to ensure the quality of the taxonomy annotations. This step was critical for removing any sequences with taxonomic discrepancies, misclassifications, or incomplete annotations that could negatively impact the correct identification of PPB. The curation process is divided into three key steps, each implemented through specific functions in the *database_filter* module (“2_run_db_filters.py”):

*Genus-Species Correspondence Check*- This is the most important step in the curation process. When importing the sequences coming from repositories such as NCBI, SILVA curators verify their correct taxonomical assignment. If discrepancies are observed between the original taxonomy and the phylogenetic assignment in the SILVA tree, SILVA curators correct the taxonomy until the genus level but conserve the original genus-species pair at the species level (see examples in Table 2). This would result in wrongly affiliated PPB during the detection process or even worse in false positive PPB. This curation step ensures that not only instances of these discrepancies observed in the MBPD [8] database are now systematically corrected, but also their sequences are accurately aligned to the pathogenic sequences before being presented in the NEMESISdb dataset.

Hence, the first step of our curation process involved the identification of discrepancies when the genus in the taxonomy did not match the genus derived at the species level. The input for this step consisted of the FASTA files produced from the *database_builder* analysis. Discrepancies and unique taxonomies with mismatches are flagged (i.e.; marked for further revision) in two Python lists for the subsequent curation step.

*Multiple-Genera Check* - The second curation step assessed multiple genera mentioned within a single taxonomic description. For example, taxonomies that included multiple genera, such as *Hafnia-Obesumbacterium or Shigella-Escherichia*, were reviewed (Table 3, Table 4). If one of the genera of the genus level matched with the genus at the species level, the taxonomy was retained; otherwise, an Excel file, “Tax_to_manual-review_{group}.xlsx”**,** was generated with sequences flagged for further manual review due to ambiguous or missing genera.

**Table 3:** Example of discrepancies between the Genus and Species level within the SILVA 138.2 SSU Ref NR99 taxonomy. The correct taxonomy goes until the genus level indicating that the sequence belongs to the bacillus genus but the genus-species pair at the species level is incorrect.

Acc number	Kingdom	Phylum	Class	Order	Family	Genus	Species
EU146061.1.1484	*Bacteria*	*Firmicutes*	*Bacilli*	*Bacillales*	*Bacillaceae*	*Bacillus*	*Streptomyces clavuligerus*

**Table 4:** Example of multiple genera within the genus level of the taxonomy.

Acc number	Kingdom	Phylum	Class	Order	Family	Genus	Species	Decision
JMPC01000305 .1.1285	*Bacteria*	*Proteobacteria*	*Gamma-proteobacteria*	*Enterobacterales*	*Entero* *bacteriaceae*	*Escherichia-Shigella*	*Acinetobacter baumannii 42,057_5*	Flagged
HG738867.2611898.2613439	*Bacteria*	*Proteobacteria*	*Gamma-proteobacteria*	*Enterobacterales*	*Entero* *bacteriaceae*	*Escherichia-Shigella*	*Escherichia coli str. K-12 substr. MC4100*	retained

*Manual Review -* A manual review process was conducted to validate the flagged discrepancies from the ambiguous or missing genera list. This review was essential for finalizing the list of sequences to be removed from the database. Following this manual review, the final set of sequences marked for deletion was established, and these sequences were subsequently removed from the dataset. The input for this stage was the file “Tax_to_manual-review_{group}.xlsx”, and the output was **“**Tax_reviewed_{group}.xlsx”, which included the “Retained” column with values of “Yes” or “No” to indicate whether the associated taxonomy (and all sequences with the same taxonomies) would be retained or deleted from the dataset.

### 4.4 The final curated FASTA dataset

To generate the final curated and validated FASTA datasets, the database_curation module (“3_run_db_curation.py”) was implemented. The process begins by reading the input Excel file Tax_reviewed_{group}.xlsx, which indicates which taxonomic entries should be excluded. For each taxonomy marked as "No", a function retrieves the corresponding sequences from the unfiltered FASTA files produced by the *database_builder* module (CRUSTACEAN_Pathogen_DB_Unfiltered.fasta, FISH_Pathogen_DB_Unfiltered.fasta, and HUMAN_Pathogen_DB_Unfiltered.fasta) to identify and remove the corresponding sequences.

As a result, the script outputs the final curated FASTA files—CRUSTACEAN_Pathogen_DB.fasta, FISH_Pathogen_DB.fasta, and HUMAN_Pathogen_DB.fasta—which include only the sequences retained after the curation process.

Additionally, at the end of this module, a species-level summary is generated for each group. An Excel file is created (Species_match_CRUSTACEAN.xlsx, Species_match_FISH.xlsx, Species_match_HUMAN.xlsx) listing the currently accepted scientific names along with all synonyms or variant names found in the database that correspond to each accepted species. This provides a reliable reference for analyzing the species composition of the curated dataset.

**Fig. 3:**
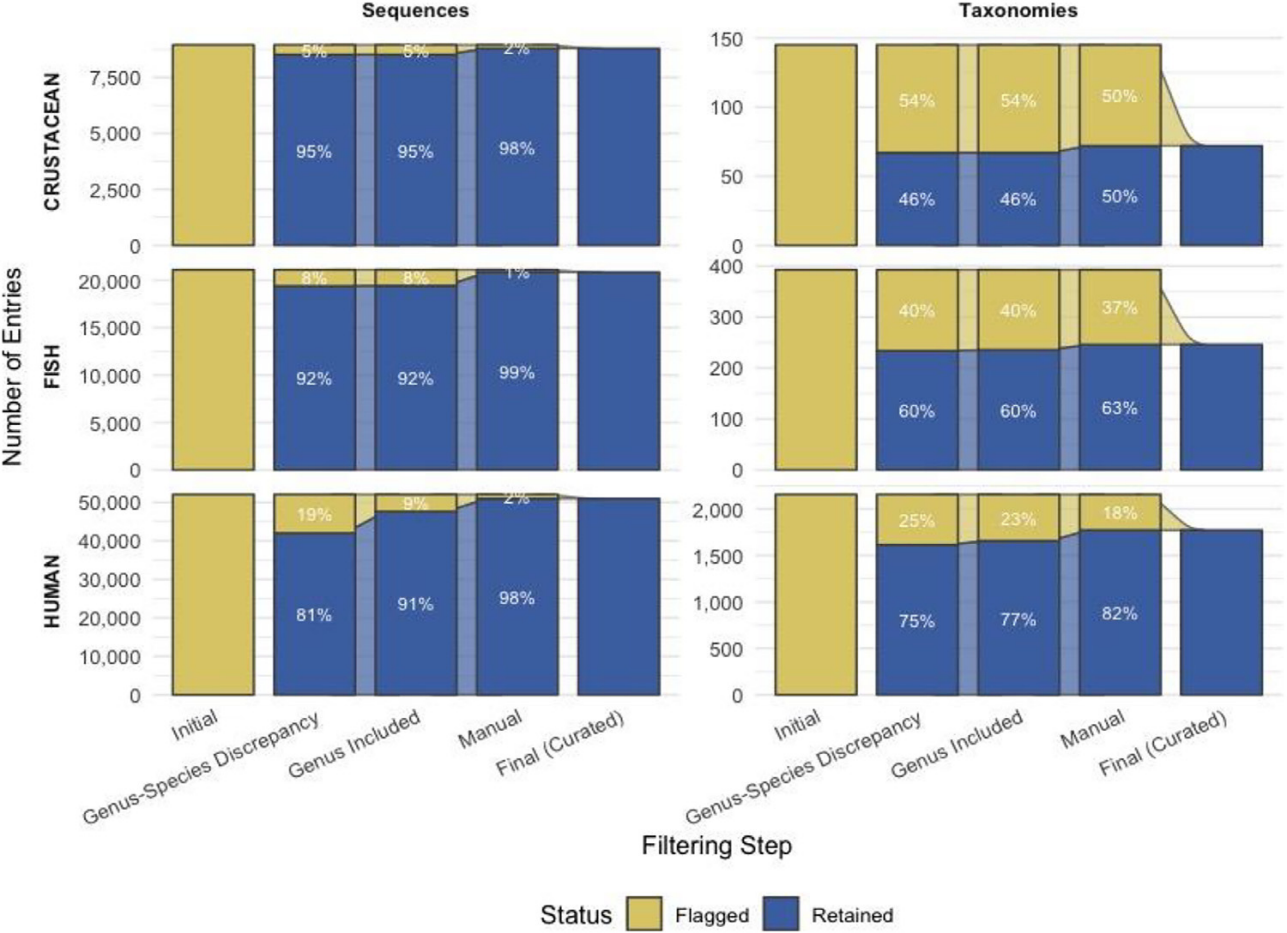
Retention and flagging of sequences and taxonomies across curation filters for each host group. Each alluvial plot shows the evolution of the number of entries (sequences or taxonomies) that were retained or flagged during the successive data curation steps. The top panels display results for sequence entries, while the bottom panels show taxonomic entries. Rows correspond to different host groups (CRUSTACEAN, FISH, HUMAN), and Y-axis scales are adapted to each case.

**Fig. 4:**
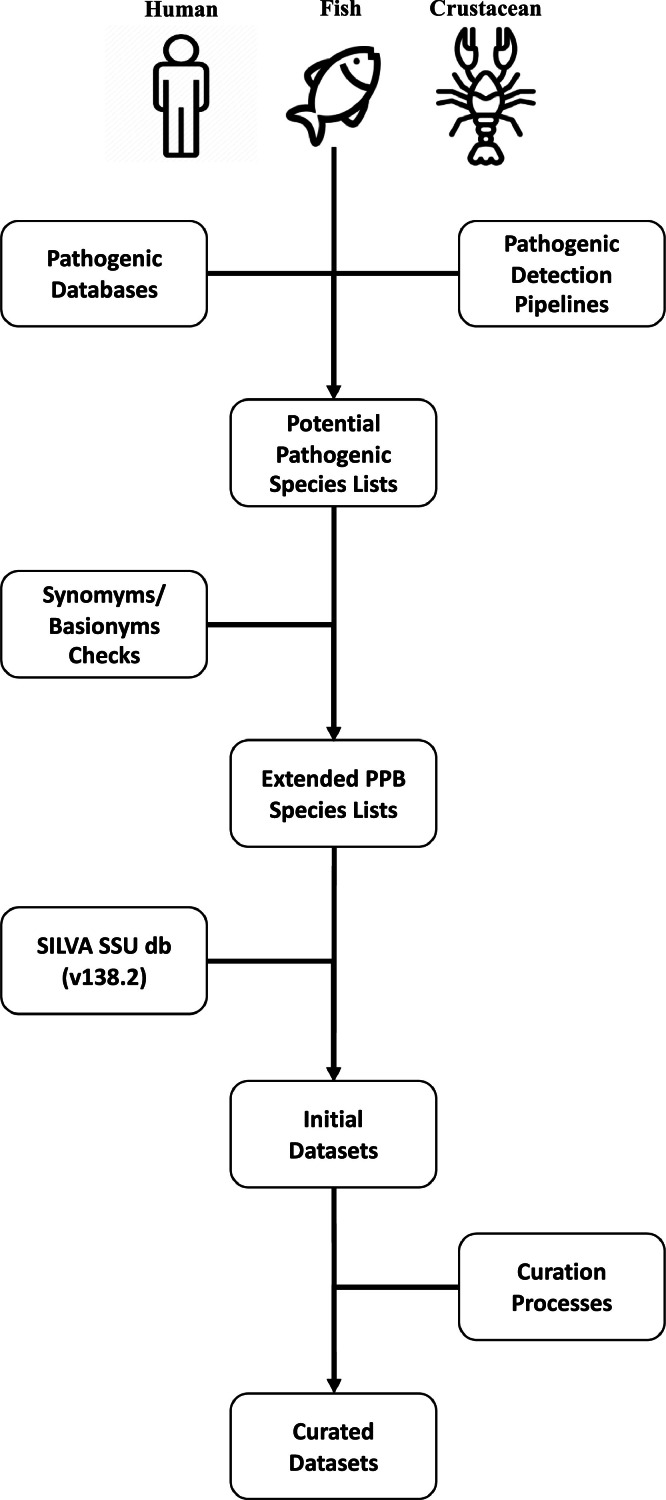
Pipeline of the creation and curation of the dataset.

Throughout the entire curation process of the datasets, the number of sequences and unique taxonomies that passed through each filter was meticulously recorded. This tracking allowed for a comprehensive understanding of the sequences and taxonomies to be eliminated for each host dataset (Fig. 3). Overall, this plot highlights how the filtering process progressively reduces the pool of sequences and taxonomies marked for elimination, leaving only a small set of sequences (i.e.; 161, 269 and 1098 respectively for Crustacean, Fish and Human) and unique taxonomies (i.e.; 73, 147 and 385 respectively for Crustacean, Fish and Human) to be removed after the final "Manual Review". Overall, the pipeline for creating and curating the dataset is briefly described in Fig. 4.

## Limitations

While amplicon and metagenome sequencing have been used to analyse the composition and risk of pathogen contamination [[14], [15], [16]], establishing the definitive pathogenicity of a bacteria still demands additional experimental validations.

## Ethics Statement

Authors have read and follow the ethical requirements for publication in Data in Brief. Authors confirm that the current work does not involve human subjects, animal experiments, or any data collected from social media platforms.

## CRediT authorship contribution statement

**Son-Hoang Tran:** Formal analysis, Investigation, Data curation, Writing – original draft. **Claudia Ximena Restrepo-Ortiz:** Methodology, Software, Data curation, Validation, Writing – review & editing. **Dinh Quang Vu:** Data curation. **Marc Troussellier:** Conceptualization, Writing – review & editing. **Yvan Bettarel:** Writing – review & editing. **Thierry Bouvier:** Writing – review & editing. **Van Ngoc Bui:** Writing – review & editing. **Nguyen Hieu Minh:** Data curation. **Trung Du Hoang:** Writing – review & editing. **Quang Huy Nguyen:** Conceptualization, Writing – review & editing. **Jean-Christophe Auguet:** Conceptualization, Methodology, Data curation, Writing – review & editing, Supervision, Project administration, Funding acquisition.

## Data Availability

ZenodoNEMESISdb (Original data)
